# Sexual and Reproductive Health Services Utilization and Associated Factors among College Students at West Arsi Zone in Oromia Region, Ethiopia

**DOI:** 10.1155/2020/3408789

**Published:** 2020-08-25

**Authors:** Demelash Wachamo, Yadessa Tegene, Abdisa Tibeso, Assefa Washo

**Affiliations:** ^1^Department of Public Health, Research and Community Service, Paradise Valley College, West Arsi Zone, Oromia, Ethiopia; ^2^Paradise Valley College, West Arsi Zone, Oromia, Ethiopia; ^3^Department of Public Health Head, Paradise Valley College, West Arsi Zone, Oromia, Ethiopia

## Abstract

**Background:**

Young people are challenged with sexual and reproductive health (SRH) problems due to limited access to services and information. It contributes a high burden of sexually transmitted disease, unsafe abortion, and premature mortality, especially in low-income countries like Ethiopia. Hence, this study aimed at assessing the SRH service utilization and associated factors among college students at West Arsi Zone in Oromia region, Ethiopia.

**Methods:**

Institutional-based cross-sectional study was conducted among 519 randomly selected college students from January 01, 2019, up to April 30, 2019. Data were collected through self-administered pretested questionnaires. Data entry and analysis for descriptive and logistic regression models were performed by using SPSS (version 23). The result was declared as statistically significant at *p* < 0.05.

**Results:**

The utilization of at least one SRH service in the last twelve months was seen in 304 (58.6%) students. Mainly information and counseling (81.3%), voluntary testing and counseling for HIV (80.6%), condom service (37.2%), pregnancy test (35.5%), family planning services (33.2%), and abortion care services (26.0%) were most frequently utilized. The students' aged between 20 and 24 years [AOR = 2.05, 95% CI: 1.38–3.06], female students [AOR = 1.92, 95% CI: 1.30–2.82], those not living with family [AOR = 1.54, 95% CI: 1.05–2.26], those who discussed about SRH with family/friends [AOR = 1.92, 95% CI: 1.31–2.82], and those who participated in school clubs [AOR = 1.75, 95% CI: 1.19, 2.58] more frequently used SRH service compared with their counterparts.

**Conclusions:**

The utilization of SRH services was low when compared with other studies and needs improvements to tackle RH-related problems. It requires pertinent health education and the SRH club at higher educational institutions is crucial.

## 1. Introduction

There were 1.2 billion youth aged 15–24 years globally in 2015 [1], youths constitute 18% of the world's population, and nearly 80% live in developing countries and 33.8% of the Ethiopian total population [2, 3]. Globally, there are 1.8 billion young people with over 80% unmet reproductive health needs in rural areas [4]. Particularly, in sub-Saharan Africa, sexual and reproductive health needs among the youth remain as a problem for young people. They are more at risk of experiencing sexual and reproductive health problems like teenage pregnancy, early marriages, sexually transmitted infections (STIs) including HIV/AIDS, and unsafe abortions [5, 6]. The college students are also vulnerable to persistent reproductive health problems [7, 8], which are related to unprecedented social, economic, and cultural change regardless of health education, family support, and health knowledge factors [9, 10].

The reproductive health complications like childbearing among young people remain high in some developing regions, as WHO estimates globally, in 2018, there were an estimated 12.8 million births among adolescent girls aged 15–19 years, representing 44 births/1000 in 2015–2020, while the highest was 99 births/1000 in the African Region [11]. Globally, 21% of women aged between 20 and 24 years reported that they were married or in an informal union before the age of 18 [12]. Across sub-Saharan African countries, 10–20% of 15- to 24-year-old report having first sexual intercourse before the age of 15 years [6]. Condom use remains under 50% [13].

In Ethiopia, national policies have created an enabling environment to improve adolescent sexual and youth-friendly reproductive health services, but the implementation remains a problem due to lack of confidentiality, lack of basic skills, and unemployment, and young women aged between 15 and 24 are the most disadvantaged [14]. Another major health threat affecting young people was reproductive tract infections including HIV/AIDS. About 26% of the HIV-positive population in Ethiopia is aged between 10 and 24 years [15, 16]. A few studies conducted on college/university students show that there is a gap in the utilization of reproductive health services with around 32% in Bahir Dar town, [17], 37.2% in Goba Town [18], and 80.5% in Madawalabu University students, Southeast Ethiopia [19]. The college/university environment increases the susceptibility of its community for HIV infection as a result of HIV high-risk behaviors and lack of parental controls [19]. Inadequate utilization of RH service has a negative effect on the health of newborn children and on the health of the young mothers and pregnant young college students, who may encounter stigma and stress and thus be less likely to complete schooling [11]. Hence, this study aimed at assessing reproductive health service utilization specifically VCT, modern contraceptives and STI diagnosis, and treatment service and its determinant factors. The finding provides local evidences discussed above and implies that young college students' health problems were related to their utilization of RH service either directly or indirectly. This study finding is important for health officials, clinicians, and health planners requiring evidence-based strategies for possible future interventions, in order to strengthen national responses to the HIV epidemic, to reduce early and unintended pregnancies, and to tackle reproductive health problems. In addition to this, the finding can be used as preliminary data for researchers and health officials to design appropriate RH programs to address all young college students' health problems.

## 2. Methods

### 2.1. Study Setting and Design

This study was conducted in four randomly selected colleges, which are in West Arsi Zone, Arsi Negele, and Shashamane Town. Four colleges were selected out of twelve colleges (Paradise Valley, Rift Valley, Pharma, and Africa Beza). They had about 19,592 students at the age of 17 up to 24 years, with 8307 male and 11285 female students (according to the college registrar, 2018). The source of population was all students. All regular college students were considered as the study population. All randomly selected regular students enrolled in the 2018 academic year for the regular program, attending their class at selected college within the study period from January 01, 2019, up to April 30, 2019, were included in the study. However, night and weekend class students were excluded from the study.

### 2.2. Sampling

The sample size was determined using single population proportion formula based on the following assumptions: (*p*) utilization of SRH service was 80.5% in Madawalabu University students, Southeast Ethiopia [19], with 95% CI (1.96), 5% margin of error (*d*), 2 design effects, and addition of 15% contingency. The final sample size was 554. Thirty percent of 12 colleges (4 colleges) was selected. At the second stage, each college was stratified into departments and 30% of total departments was selected using simple random sampling. Finally, the estimated sample size was allocated to each department proportionally to size, and participants were selected by using simple random sampling from students list of each selected section.

### 2.3. Data Collection Tools and Procedures

Data were collected through self-administered pretested questionnaires. The utilization of SRH services withinthe last 12 months, at any service providing health institution. This was measured through the dichotomous response (yes/no). The positive (“yes”) response was further validated with questions on the type of SRH services utilized (information/counseling on SRH issues, family planning, voluntary testing, counseling on HIV, abortion care, and testing and treatment of STIs). The questionnaire was developed by collecting and adopting after customizing into the study context from various literatures [20–22]. The quality of data was assured by translating questionnaires from English to Afan Oromo and then back to English by another expert using properly designed and pretested questionnaire. The data were collected by trained 4 diploma nurses and supervised by 2 BSc nurses. The pretest was done on 5% of the sample size outside the study area, and some modifications on sequence and arrangement of multiple answer questionnaire were made. To maintain confidentiality, each participant took a single sparsely arranged seat, and the participant put the filled questionnaire on a locally prepared cartoon box which was arranged at the corner of the room. Filled questionnaires were collected after checking for consistency and completeness.

### 2.4. Data Analysis

Data entry, cleaning, and analysis were done by SPSS (version 23). Descriptive analysis including frequency distribution and the percentage was used to determine the utilization of SRH services and to describe sociodemographic and other individual-related variables. All factors with a *p* value <0.25 in the bivariate logistic regression analysis were a candidate to the multivariable model to control confounding effects. The Hosmer–Lemeshow goodness-of-fit statistic was used to assess whether the necessary assumptions for the application of multiple logistic regression are fulfilled. Odds ratios (OR) with 95% confidence intervals (CIs) were calculated. Finally, a *p* value <0.05 declared a significant association.

### 2.5. Operational Definitions

#### 2.5.1. Youth

World Health Organization (WHO) defines youth as those between the ages of 15 and 24 years [23].

#### 2.5.2. Utilization of AYRH

It was assessed based on youths visited, and usage of any one of the services from preexisting YFS facilities of Hawassa city within the past twelve months was regarded as service utilization [18].

#### 2.5.3. Youth-Friendly Reproductive Health Services

In this study, services such as family planning, VCT, STI diagnosis and treatment, abortion care, postabortion care, and condom use are available within YFS clinics. Finally, youth-friendly services utilization was considered if the respondent utilizes one of these services in the last 12 months.

## 3. Results

### 3.1. Sociodemographic Characteristics

A total of 519 college students were enrolled in the study with a response rate of 93.7%. The average age of the students was 20.4 (±standard deviation (SD), 1.84) years and 338 (65.1%) in the age of 20–24 years. Out of the participants, (59.9%) females, (55.7%) were previously urban dwellers and (52.0%) were living with family and 271 (52.2%) were earning below 300 pocket money in Ethiopian Birr (ETB) per month. Majority of the participants (94.6) were single in this study. Regarding student's family educational status, 24.1% mothers and 6.9% fathers had no formal education, while only 15.0% mothers and 39.5% fathers had attended college and received higher education (Table 1).

### 3.2. Knowledge of SRH Services and Sexual Practices

**Figure 1:**
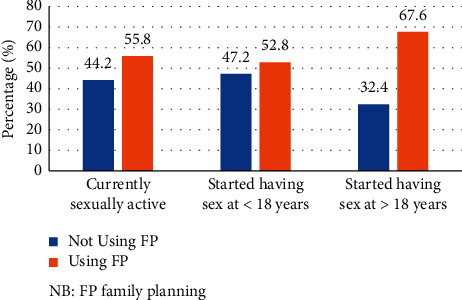
Utilization of family planning services among sexually active college students in West Arsi Zone, Oromia, Ethiopia, 2019 (*n* = 186). FP = family planning.

Almost 515 (99.2%) students have heard about services provided under SRH services. Their main source of information was heath providers (37.5%), school (28.2%), and peers (13.2%). The majority of the participants aware of health facilities where to get SRH services (86.5%), the right to use RH services (71.0%), the method of family planning (92.8%), and know signs and symptoms of STI (77.8%) were identified correctly. About half of the participants reported discussion with family or friends (49.7%), participated in school clubs (52.0%), and know modes of transmission of STI was unprotected sex (57.7%) (Supplementary Table S1). Out of 519 students, 46.6% had experienced sex; among this, 200 (82.6%) had their first sex before the age of 18 years. About one-third of the students (35.8%) were currently sexually active; among this, only 55.8% have used any one of the family planning methods in the last twelve months (Figure 1).

### 3.3. Utilization of SRH Services

**Figure 2:**
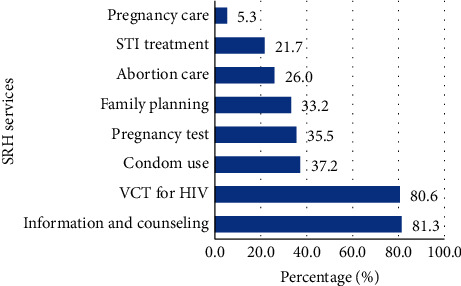
Utilization of SRH services among college students in West Arsi Zone, Oromia, Ethiopia, 2019 (*n* = 304). STI = sexually transmitted infection; VCT = voluntary counselling and testing.

The utilization of at least one SRH services in the last twelve months was seen in 304 (58.6%) students [95% CI: 54.3–62.8]. Among this, mainly information and counseling on SRH services (247 (81.3%)), VCT for HIV (245 (80.6%)), condom service (113 (37.2%)), pregnancy test (108 (35.5%)), family planning services (101 (33.2%)), and abortion care services (79 (26.0%)) were most frequently utilized SRH services (Figure 2). The major reasons for not using SRH services was the distance to the health facility (104 (48.4%)), preferred sex of SRH service provider (109 (50.7%)), and working hour of the health facility (114 (53.0%)), as reported by about half of students (Supplementary Figure S1).

### 3.4. Associated Factors of the Utilization of SRH Services

In the multivariate analysis, the age of students, sex, living arrangement, discussing SRH with family/friends, and participation in school clubs remained as the determinant of at least one SRH service. Students in the age of 20–24 years 2 times more frequently used SRH services [AOR = 2.05, 95% CI: 1.38–3.06] compared with younger age. Also, female students [AOR = 1.92, 95% CI: 1.30–2.82] more frequently used SRH services than males. The students not living with family [AOR = 1.54, 95% CI: 1.05–2.26], those who discussed about SRH services with family/friends [AOR = 1.92, 95% CI: 1.31–2.82], and those who participated in school clubs [AOR = 1.75, 95% CI: 1.19, 2.58] more frequently used SRH services compared with counterparts (Table 2).

## 4. Discussion

This institution-based cross-sectional study revealed that the utilization of at least one SRH service in the last twelve months was seen in 58.6% [95% CI: 54.3–62.8]. Mainly information and counseling (81.3%), voluntary testing and counseling for HIV (80.6%), condom service (37.2%), pregnancy test (35.5%), family planning services (33.2%), and abortion care services (26.0%) were most frequently utilized. The result also showed 55.8% of the currently sexually active respondents had utilized family planning methods in the last twelve months. This study result indicates that there is still unmet need of students.

This study result was consistent with the study findings in 59.2% among Aksum University students, Shire Campus, Shire Town, Tigray, Ethiopia [24], and 54.7% of them had utilized at least one reproductive health service among the youth in Amhara Region, Ethiopia [25]. This result is higher compared to that in Hadiya Zone, Ethiopia (35.0%) [26] and Sodo, Southern Ethiopia (40.6%) [27] and lower than from in Madawalabu University students, Southeast Ethiopia (80.5%) [19], and 67.2%were advised about sexual and reproductive health SRH service at Southwest Oromia, Ethiopia [28]. The difference in findings may be due to the implementation of SRH service various in college and high school community. In addition to this, it could be explained as variations in the socioeconomic and cultural factors of the study participants.

This study result revealed that older age students more frequently used SRH services than those in younger age. This finding was similar to the study reported in Northern region, Ghana [29], and in Hong Kong, China [30]. This may be because students with younger age lack sexual experience and SRH service information and health benefits. Furthermore, female students more frequently used SRH services than males. It agrees with a study conducted in Hong Kong, China [30], and Tanzania [31]. This could be justified that female students utilized more SRH services because they naturally had high exposure to sexually transmitted infections including HIV/AIDS and had unwanted pregnancy.

This study result shows that the students who were not living with the family more frequently use SRH services than students who live with their family. This finding was consistent within Nekemte Town, Ethiopia [15], Australia [32], and Lao People's Democratic Republic [3]. This explains that living with family and being under parental control may protect students from early sexual exposure. In addition to this, the students who discussed SRH services with family/friends more frequently used SRH services than their counterparts, as reported similarly in Nekemte town, Ethiopia [15], Woldia town, Northeastern Ethiopia [33], and Selangor, Malaysia [34]. This can be justified by the fact that discussion of services with parents and friends favors youths to exchange information and experiences that allow them to use the services.

This study result shows that the students who participated in school clubs had a significant association with the utilization of SRH services. This finding was also agreed with the study conducted in Adet Tana Haik College students, Northwest Ethiopia [35], Wolaita Sodo, Southern Ethiopia [36], and Debre Markos town, Northwest Ethiopia [37]. This explains that school clubs allow to communicate and share ideas, knowledge, and experiences with their friends on sexual and reproductive health issues that affect the utilization of the service.

This study result shows there was low utilization of SRH services in the last twelve months among college students compared with other studies. This was worsened by perceived barriers like a long distance from a health facility, preferring specific sex of SRH service provider due to cultural factors, lack of separate room for service, and incontinent working hours of the health facility. This implies that a lot has to be done on awareness creation about the nature of YFS and for supporting the youth so that they could pay due attention to improve SRH services and make condom services available for users at any time including weekend.

### 4.1. Limitations

There might be a social desirability bias on personal and sensitive issues, and obtaining honest responses among young students might have been difficult and is a limitation of this study although we tried to collect data by self-administered questioner with collection in locally prepared boxes to ensure their privacy. Hence, the quantitative study design did not allow for probing into certain areas that needed further qualitative description. This study was conducted in one college, and the findings may not be generalizable to all adolescents and youth populations in Ethiopian. In addition to this, the odds ratios of the cross-sectional study did not show the strength of an association.

## 5. Conclusions

This study result shows that there was low utilization of SRH services in the last twelve months among college students when compared with other studies. The information and counseling about SRH services, voluntary testing and counseling for HIV, condom service, pregnancy test, family planning services, and abortion care services were most frequently utilized. The older age students, female, not living with family, those who discussed on SRH with family/friends, and those who participated in school clubs more frequently used SRH services compared with their counterparts. The public health officials, concerned organizations, and clinicians need to focus on the utilization of SRH services among college students by providing mini media programs on information and counseling on SRH services like VCT, condom service, and safe sex. The colleges and West Arsi Zone health departments need to provide pertinent health education on utilization of SRH services at higher educational institutions. In addition to this, capacitate the SRH club and collaboration with parents to improve SRH services utilization at higher educational institutions. It is possible and less cost to implement by collaborating with West Arsi Zone health departments by introducing new strategies like providing training for volunteer students on SRH services, availing free condoms, and establishing health clubs and mini media programs at studied colleges.

## Figures and Tables

**Table 1:** Sociodemographic characteristics of college students in West Arsi Zone, Oromia, Ethiopia, 2019.

	Category	No.(%)
Age	15–19 years	181 (34.9)
	20–24 years	338 (65.1)
Sex of respondent	Male	208(40.1)
	Female	311 (59.9)
Previous residence	Urban	289 (55.7)
	Rural	230 (44.3)
Living arrangement	Living with family	270 (52.0)
	Not living with family	249 (48.0)
Year of study	First year	191 (36.8)
	Second year	169 (32.6)
	Third year	159 (30.6)
Pocket money in ETB	Below 300	271 (52.2)
	300–900	202 (38.9)
	900 and above	46 (8.9)
From where do you get money	Pocket money only	53 (10.2)
	Family	319 (61.5)
	Relative	26 (5.0)
	Friend	22 (4.2)
	Per time work	84 (16.2)
	Others	15 (2.9)
Mother's education	No formal education	125 (24.1)
	Primary school	199 (38.3)
	High school	117 (22.5)
	College and above	78 (15.0)
Father's education	No formal education	36 (6.9)
	Primary school	141 (27.2)
	High school	137 (26.4)
	College and above	205 (39.5)
Marital status of students	Single	491(94.6)
	Married	24(4.6)
	Separate	4(0.8)

ETB = Ethiopian birr.

**Table 2:** Bivariable and multivariable logistic regression analysis for utilization of SRH service among college students in West Arsi Zone, Oromia, Ethiopia, 2019.

		Utilized of SRHS		Corollary (95% CI)	AOR (95% CI)	*p* value
		Yes Number (%)	No Number (%)			
Age	15–19 years	82 (45.3)	99 (54.7)	1	1	
	20–24 years	222 (65.7)	116 (34.3)	2.31 (1.60, 3.34)	2.05 (1.38, 3.06)	<0.001^*∗*^
Sex	Male	96 (46.2)	112 (53.8)	1	1	
	Female	208 (66.9)	103 (33.1)	2.36 (1.64, 3.38)	1.92 (1.30, 2.82)	0.001^*∗*^
Previous residence	Rural	121 (52.6)	109 (47.4)	1	1	
	Urban	183 (63.3)	106 (36.7)	1.56 (1.09, 2.21)	1.46 (1.00, 2.15)	0.052
Living arrangement	Living with family	144 (53.3)	126 (46.7)	1	1	
	Not living with family	160 (64.3)	89 (35.7)	1.57 (1.11, 2.24)	1.54 (1.05, 2.26)	0.027^*∗*^
Year of study	First year	100 (52.4)	91 (47.6)	1	1	
	Second year	104 (61.5)	65 (38.5)	1.46 (0.96, 2.22)	1.48 (0.93, 2.34)	0.096
	Third year	100 (62.9)	59 (37.1)	1.54 (1.00, 2.37)	1.49 (0.93, 2.39)	0.097
Discussed about SRH with family/friends	No	129 (49.4)	132 (50.6)	1	1	
	Yes	175 (67.8)	83 (32.2)	2.161 (.51, 3.08)	1.92 (1.31, 2.82)	0.001^*∗*^
Participated in school clubs	No	127 (51.0)	122 (49.0)	1	1	
	Yes	177 (65.6)	93 (34.4)	1.83 (1.28, 2.60)	1.75 (1.19, 2.58)	0.005^*∗*^
Mother's education	No formal education	63 (50.4)	62 (49.6)	1	1	
	Primary school	119 (59.8)	80 (40.2)	1.46 (0.93, 2.30)	1.61 (0.98, 2.62)	0.058
	High school & above	122 (62.6)	73 (37.4)	1.65 (1.04, 2.59)	1.63 (0.99, 2.68)	0.053

^*∗*^Statistically significant on multivariate analysis *p* value (<0.05); COR: crude odds ratio; AOR: adjusted odds ratio; CI: confidence interval, 1: reference, SRHS = sexual and reproductive health services; SRH = sexual and reproductive health issues.

## Data Availability

There are no remaining data and materials, and all information is presented in the main manuscript. The questionnaire used for this study is available as “Additional file 2-Self-administered questioner,” and the raw data used for the statistical analysis are indicated as “Additional file 1.”

## Abbreviations

ANC: Antenatal care
AOR: Adjusted odds ratio
COR: Crudes odds ratio
ETB: Ethiopian birr
RH: Reproductive health
SPSS: Statistical package for social science
SRH: Sexual and reproductive health
SRHS: Sexual and reproductive health services
STIs: Sexually transmitted infections
YFRHS: Youth-friendly reproductive health services
YFS: Youth-friendly services
WHO: World Health Organization.

## Supplementary Materials

Supplementary Materials Supplementary Figure S1: the reasons for not utilizing SRH services among college students in West Arsi Zone, Oromia, Ethiopia, 2019 (*n* = 215). Supplementary Table S1: utilization of SRH services among college students in West Arsi Zone, Oromia, Ethiopia, 2019. Additional file 1 shows the data used for statistical analysis (CSV, 60.2 kb). Additional file 2 shows a self-administered questioner to assess the SRH services and associated factors among college students (doc., 75.5 kb).
