# Supplementary material for: Sexual and Reproductive Health Services Utilization and Associated Factors among College Students at West Arsi Zone in Oromia Region, Ethiopia
Source: ScientificWorldJournal. 2020 Aug 25;2020:3408789. doi: 10.1155/2020/3408789 (PMC7468662; doi:10.1155/2020/3408789)
Supplement: Supplementary Materials — Supplementary Figure S1: the reasons for not utilizing SRH services among college students in West Arsi Zone, Oromia, Ethiopia, 2019 (n = 215). Supplementary Table S1: utilization of SRH services among college students in West Arsi Zone, Oromia, Ethiopia, 2019. Additional file 1 shows the data used for statistical analysis (CSV, 60.2 kb). Additional file 2 shows a self-administered questioner to assess the SRH services and associated factors among college students (doc., 75.5 kb). [file 3408789.f1.zip › 3408789.f1/Additional file 2 -Self-administered questioner.doc]

## Self-administered questioner

A self-administered questioner prepared to assess the SRH services among College Students in West Arsi Zone, Oromia, Ethiopia.

**PART I: Socio-demographic and socioeconomic characteristics**

**Direction:** circle the numbers for response categories	Code______

| **S.No.** | **Questions** | **Coding categories** | **Skip** |
| --- | --- | --- | --- |
| 101 | Age in year | **________** |  |
| 102 | Sex of the respondent | 1. Male 2. Female |  |
| 103 | Your Place of origin | 1. Urban 2. Rural |  |
| 104 | With whom you are livings? | 1. With family 2. Private on rented house |  |
| 105 | Year of study | 1. First year 2. Second year 3. Third year |  |
| 106 | What is your monthly income in Ethiopian Birr | _________ |  |
| 107 | From where do you get money? | 1. Pocket money only 2. Family 3. Relative 4. Friend 5. Per time work 6. Others |  |
| 108 | Mother's education | 1. No formal education 2. primary school 3. High school 4. College and above |  |
| 109 | Father's education | 1. No formal education 2. primary school 3. High school 4. College and above |  |
| 110 | Mother's main occupation | 1. Employed 2. Merchant 3. House wife |  |
| 111 | Father's occupation | 1. Unemployed 2. Employed 3. Merchant |  |
| 112 | What is your marital status | 1. Single 2. Married 3. Divorced 4. Widowed 5. Separate |  |

**Part two. Knowledge** about reproductive health services

| Q.No**.** | Questions | Coding Categories’ |  |
| --- | --- | --- | --- |
| 201 | Have you ever heard about youth friendly reproductive health service? | 1. Yes  0. No ______________skip to | **300** |
| 202 | Which services are provided under YFRHS?  (can mark more than one response) | 1. Family planning 2. Treatment of sexually transmitted infection 3. Voluntary counseling and testing for HIV/AIDS 4. Abortion service 5. Antenatal care 6. Information education and communication 7. Condom use 8. Others (specify)_____________ |  |
| 203 | If yes, for question number 300, from where you got information?  (can mark more than one response) | 1. Parents 2. School 3. Heath providers 4. Peers 5. Posters 6. Radio 7. Others (specify)_____________ |  |
| 204 | Have you ever discussed about RH issues with parents/guardians? | 1. Yes  0. No |  |
| 205 | Do you know where you get youth friendly services? | 1. Yes  0. No |  |
| 206 | where you get youth friendly services? | 1. Health center 2. Drug shop 3. Traditional healer 4. Others (specify)_____________ |  |
| 206 | Youths/ adolescents have the right to use RH services? | 1. Yes  0. No |  |
| 207 | Do you know any family planning method? | 1. Yes  0. No __________________ skip to | 208 |
| 208 | Which of the following family planning methods do you know? (choosing more than one answer is possible) | 1. condom 2. implant 3. pills 4. IUCD  5. Injectable 7. other |  |
| 209 | Have you ever heard about sexually transmitted infections? | 1. Yes  0. No _________________ skip to | **300** |
| 210 | Which of the following lists do you know? | 1. Syphilis 2. Gonorrhea  3. Chancroid 4. HIV |  |
| 211 | Do you know any sign and symptoms of sexually transmitted infections? | 1. Yes  0. No |  |
| 2012 | What sign and symptoms of sexually  transmitted infections? | 1. Genital ulcer 2. Genital discharge  3. Itching 4. Burning urination 5. others |  |
| 2013 | What modes of transmission of sexually transmitted infections do you know? | 1. Contact with infectious blood 2. Mother to child 3. Unprotected sex 4. Using sharp materials with an infected person 5. Others |  |

**Part three: Individual factors** on Health institution-based factors

| Q.No | Questions | Coding Categories | **Skip** |
| --- | --- | --- | --- |
| 300 | Is there a YFRHS in your living area? | 1. Yes 2. No ________________skip to | 302 |
| 301 | How far is the health facility from your home? | ___________(specify in minute) |  |
| 302 | Have you ever asked to pay for the service you used? | 1. Yes 2. No |  |
| 303 | Is the working hour of the health facility convenient for you? | 1. Yes 2. No ______________ skip to | 400 |
| 304 | If No, for the above question, what would be the most convenient time for you? | 1. Earlier in the morning 2. late the afternoon 3. weekends 4. Holidays (school holidays) 5. Other (specify) |  |
| 305 | For how long do you wait to get service? | 1. Yes 2. No |  |
| 306 | Age at first sexual intercourse | 1. < 18 years 2. ≥ 18 years |  |

Part four: Questions about utilization of youth friendly service

| **Q. No** | **Question** | **Coding Categories** |  |
| --- | --- | --- | --- |
| 400 | Have you ever used any RH service in the past twelve months? | 1.yes  2. No________________ skip to | **403** |
| 401 | What services did you use in the past twelve months?  (ticking more than one response is/are possible) | 1. Family planning 2. STD treatment 3. VCT for HIV/AIDS 4. Abortion service 5. Post abortion services 6. ANC 7. Condom use 8. Information, education & counseling 9. Other services (specify)_________ |  |
| 402 | From where did you use RH service in the past twelve months? | 1. Youth center 2. Hospital 3. Health center 4. Family guidance association 5. Other (specify) |  |
| 403 | If no why?  (tick more than one response is possible) | 1. Distance to the health facility 2. Don't know where to go 3. Fear of being seen by parents or people 4. whom they knew 5. Cost of service 6. Others (specify) |  |
