# Supplementary material for: Sexual and Reproductive Health Services Utilization and Associated Factors among College Students at West Arsi Zone in Oromia Region, Ethiopia
Source: ScientificWorldJournal. 2020 Aug 25;2020:3408789. doi: 10.1155/2020/3408789 (PMC7468662; doi:10.1155/2020/3408789)
Supplement: Supplementary Materials — Supplementary Figure S1: the reasons for not utilizing SRH services among college students in West Arsi Zone, Oromia, Ethiopia, 2019 (n = 215). Supplementary Table S1: utilization of SRH services among college students in West Arsi Zone, Oromia, Ethiopia, 2019. Additional file 1 shows the data used for statistical analysis (CSV, 60.2 kb). Additional file 2 shows a self-administered questioner to assess the SRH services and associated factors among college students (doc., 75.5 kb). [file 3408789.f1.zip › 3408789.f1/Supplementary Figure S1.doc]

Figure S1: The reasons for not utilizing YFRHS among College Students in West Arsi Zone, Oromia, Ethiopia, 2019. (n=215)
