# Supplementary material for: Sexual and Reproductive Health Services Utilization and Associated Factors among College Students at West Arsi Zone in Oromia Region, Ethiopia
Source: ScientificWorldJournal. 2020 Aug 25;2020:3408789. doi: 10.1155/2020/3408789 (PMC7468662; doi:10.1155/2020/3408789)
Supplement: Supplementary Materials — Supplementary Figure S1: the reasons for not utilizing SRH services among college students in West Arsi Zone, Oromia, Ethiopia, 2019 (n = 215). Supplementary Table S1: utilization of SRH services among college students in West Arsi Zone, Oromia, Ethiopia, 2019. Additional file 1 shows the data used for statistical analysis (CSV, 60.2 kb). Additional file 2 shows a self-administered questioner to assess the SRH services and associated factors among college students (doc., 75.5 kb). [file 3408789.f1.zip › 3408789.f1/Supplementary Tables S1.doc]

Table S1: Utilization of youth friendly reproductive health services among College Students at Shashemene Town in West Arsi Zone, Oromia, Ethiopia, 2019.

|  | **Category** | **No.** | **(%)** |
| --- | --- | --- | --- |
| Ever heard about services provided under YFRHS. | | |  |
|  | No | 4 | (.8) |
|  | Yes | 515 | (99.2) |
| Source of information on SRH services | |  |  |
|  | parents | 60 | (11.6) |
|  | School | 146 | (28.2) |
|  | Heath providers | 194 | (37.5) |
|  | Peers | 27 | (5.2) |
|  | Posters | 22 | (4.3) |
|  | Radio | 68 | (13.2) |
| Awareness of health facilities where to get SRH services | |  |  |
|  | Health center | 447 | (86.5) |
|  | Drug shop | 58 | (11.2) |
|  | Traditional healer | 6 | (1.2) |
|  | Others (specify) | 6 | (1.2) |
| Youths have the right to use RH services | |  |  |
|  | No | 150 | (29.0) |
|  | Yes | 367 | (71.0) |
| Know any method of family planning | |  |  |
|  | No | 37 | (7.2) |
|  | Yes | 480 | (92.8) |
| Know sign and symptoms of STI | |  |  |
|  | No | 115 | (22.2) |
|  | Yes | 402 | (77.8) |
| Know sign and symptoms of sexually | |  |  |
|  | Genital ulcer | 131 | (25.2) |
|  | Genital discharge | 94 | (18.1) |
|  | Itching | 205 | (39.5) |
|  | Burning urination | 25 | (4.8) |
|  | I don't know | 64 | (12.3) |
| Know modes of transmission of sexually transmitted infections | |  |  |
|  | Contact with infectious blood | 84 | (18.4) |
|  | Mother to child | 75 | (16.4) |
|  | Unprotected sex | 263 | (57.7) |
|  | Using sharp materials with an infected person | 34 | (7.5) |
| Discussed about RHS with family/friends | |  |  |
|  | No | 261 | (50.3) |
|  | Yes | 258 | (49.7) |
| Participated in school clubs | |  |  |
|  | No | 249 | (48.0) |
|  | Yes | 270 | (52.0) |
